# Antibiotic use on German pig farms - A longitudinal analysis for 2011, 2013 and 2014

**DOI:** 10.1371/journal.pone.0199592

**Published:** 2018-07-03

**Authors:** Malin Hemme, Inga Ruddat, Maria Hartmann, Nicole Werner, Lisa van Rennings, Annemarie Käsbohrer, Lothar Kreienbrock

**Affiliations:** 1 Department of Biometry, Epidemiology and Information Processing, WHO Collaborating Centre for Research and Training for Health in the Human-Animal-Environment Interface, University for Veterinary Medicine, Hannover, Germany; 2 Federal Institute for Risk Assessment, Berlin, Germany; 3 Institute for Veterinary Public Health, Veterinary University Vienna, Vienna, Austria; The University of Melbourne, AUSTRALIA

## Abstract

To study antibiotic use in livestock in a temporal context with the development of antimicrobial resistance, long-term changes in antibiotic use must be mapped and their possible causes must be explored. Therefore, the present work assesses the changes in antibiotic use over time in German livestock husbandry. In addition, factors associated with antibiotic use were analyzed to identify possible strategies for further reducing antimicrobial usage. For 2011, 2013 and 2014, antibiotic usage data were collected and examined within the VetCAb project. Three hundred participating pig holdings provided information on their antibiotic use based on obligatory application and delivery forms (ADFs) filled in by their veterinarian as well as information on their current stabling capacities for each production type held. Data on sow, piglet, weaner and fattening pig holdings were described separately, using the semi-annual treatment frequency (TF) to measure antibiotic consumption. Multiple linear mixed models were used to investigate the effects of time, farm size, region and farm management category on the treatment frequency. The study yielded significant time changes with p-values below 0.001 in antibiotic administration with a decreasing median TF in piglets from 3.8 in the first half of 2011 (IQR = 1.1–10.6) to 1.7 in the second half of 2014 (IQR = 0.2–4.5) and in fattening pigs from 5.1 in the first half of 2011 (IQR = 0.2–15.4) to 0.7 in the second half of 2014 (IQR = 0.1–6.7). Meanwhile the TF fluctuated between 8.2 and 12.2 in weaners during the observational period (IQRs between zero (lower quartile) and 37.9 (upper quartile)). Piglet, weaner and fattening pig holdings belonging to the upper third of the holdings in size used significantly more antibiotics than the other holdings investigated. Particularly for weaner and fattening pig holdings, a higher TF was noted for farms without breeding units. The region was only a significant factor in weaners. In conclusion, for 2011, 2013 and 2014, the present study shows a clear reduction in antibiotic treatment frequency in German pig holdings. In addition, the association with various factors such as herd size and farm organization on the antibiotic usage frequency is indisputable. Therefore, these factors should be included in monitoring systems and considered when evaluating intervention measures.

## Introduction

Knowledge of antibiotic use is necessary for containing of antimicrobial resistance. In 1998, the EU invitational conference, "The Microbial Threat", issued recommendations on how to combat the increasing threat of developing antimicrobial resistance [1–3]. In 2000, the WHO emphasized the key role of antibiotic and resistance monitoring system data in controlling antimicrobial resistance in their "Global Principles for the Containment of Antimicrobial Resistance in Animals intended for Food" [4]. Recently, the European Medicines Agency (EMA) and the European Food Safety Authority (EFSA) stressed that collecting antimicrobial resistance and consumption data is key to establishing effective measures to control antimicrobial resistance [5]. The World Health Organization (WHO), the Food and Agriculture Organization of the United Nations (FAO), and the World Organization for Animal Health (OIE) presented a global action plan on antimicrobial resistance considering different integrative measures [6–8].

In recent years, various systems have been developed worldwide to map the quantity and frequency of antibiotic use in veterinary medicine. In Germany, one of these monitoring systems was developed within the VetCAb (Veterinary Consumption of Antibiotics) project, which was the first project to publish representative data on antibiotic use in German livestock husbandry. In 2007 and 2008, the VetCAb feasibility study was conducted to determine whether representative monitoring of antibiotic consumption in Germany is feasible, which data can be used as a basis and how the data collection can be organized. This study’s results were published by Merle et al. [9, 10]. The VetCAb pilot study followed, in which representative data on antibiotic use were collected throughout Germany for 2011 [11]. Thereafter, the VetCAb project was refunded, and the data have been collected from 2013 to present as the longitudinal VetCAb sentinel study.

Currently, the results from various monitoring systems show substantially reduced antibiotic use in German livestock husbandry [12–15], as well as in other European countries (for example MARAN [16], SVARM [17] and DANMAP [18]). Systems in other countries are often based on quantities determined by sales data [19], or the amount prescribed by animal species. In Germany, several monitoring systems present their results based on antibiotic usage frequency, such as the QS monitoring system [20], the nationwide official database implemented by the German Medicinal Products Act (the Federal Office of Consumer Protection and Food Safety; [21]) and the VetCAb project.

To analyze the temporal trend in more detail, the present work focused on the change in antibiotic usage over time in German livestock husbandry, investigating the years 2011, 2013 and 2014. Since some publications have shown that various factors influence both disease incidence [22–24] and antibiotic use magnitude [25], the question arises, weather specific factors associated with antibiotic use can be identified. To develop strategies for further reducing antimicrobial usage and to correct the results of the antimicrobial use frequency for other effects, these factors must be analyzed.

Thus, in the present work, the influences of temporal trends, farm size, farm category, region and the veterinarian supervising the farms on the treatment frequency are considered via regression models.

## Materials and methods

### Study design

The VetCAb sentinel study is a longitudinal extension of the cross-sectional VetCAb pilot study [11] and data for 2011, 2013 and 2014 from Germany are presented. The study population is an open cohort with ongoing recruitment of farms and veterinarians to compensate for possible withdrawals and stabilize the study size and representativeness over time. Therefore, the study population consists of farms that previously participated in 2011, and farms that were recruited later.

Every participant (farmer or veterinarian) provided information on antimicrobial substance application and/or delivery, including information on the delivery/application date, number of animals treated, name and amount of the antibiotic drug used, the medical indication and the treatment duration. Because documenting this information is mandatory for farmers and veterinarians in Germany within a five-year liability period, this information can be obtained retrospectively from the application and delivery forms (ADFs) in farming practice. Data were exported from the veterinarian’s software system, the database of the company "QS Qualität und Sicherheit GmbH", who run a private monitoring system in Germany, and from manual data entry within the study system. Furthermore, farmers provided the number of livestock places for every production type kept.

As in the pilot study, all participants provided consent to use their data for this study, given that all personal data on the pig holding farmers and veterinarians were pseudonymized, as per the privacy statement given to each participant.

All data are transferred manually or imported into a database. Interfaces to several official and private computer systems containing antibiotic use data are integrated in the system. The database is based on the open-source relational database system, MySQL and was designed exclusively for this project. Plausibility checks for completeness and pharmacological plausibility are performed during data input and obvious incorrect entries are intercepted. Subsequently, the dataset is also checked in detail regarding different focuses: Farms without antibiotic use and farms with unusually high treatment frequencies (TFs) are checked for correct information. Missing or incorrect information is revised and, if obtainable, corrected or excluded from the analyses.

In this survey, four production type groups are considered: sows (averaging 200 kg), suckling piglets (averaging 4 kg), weaners (averaging 15 kg) and fattening pigs (averaging 50 kg). The average weights were used per the pilot study [11]. Group allocation is based on the ADF sheet category. Each participating farm could hold one or more production type. Each production type group held on a single farm is defined as a holding in the analysis.

### Measuring antibiotic usage

The evaluation focuses on calculating the number of drug applications (treatment units; number of used daily doses; nUDD):

nUDD=numberofanimalstreated×numberofdaystreated×numberofactiveingredients,

as well as estimating the average number of treatments per animal (treatment frequency; TF):

$$\mathrm{TF}=\frac{\mathrm{nUDD}}{\mathrm{farm}\;\mathrm{size}}$$

[26–29]. The measurements were calculated for all applications in a holding within a six-month period (half-year).

This calculation is consistent with those used by other authors [30, 31]; however, as demonstrated in Schaekel et al. [29], most antibiotic consumption calculations use an average body weight and DDDA to calculate the nUDD. Here, ADFs include this information directly.

In this analysis, the number of livestock places is used as the population at risk to calculate the TF, i.e., the population size refers to the possibility of keeping animals rather than the number of animals kept [11, 26]. As livestock places for piglets are not observed directly, the number of livestock places for sows is multiplied by 10.25, the average number of piglets per litter in Germany per Frisch et al. [32].

### Statistical analysis

To study the association of various factors with antibiotic use, a multiple linear regression model is performed separately for each defined production type group separately using TF as the outcome. For this purpose, a right-trimmed data set was used to guarantee robust model estimators, where the top 1% of TFs are excluded [33].

The explanatory variable set in each model contains "Time", "Farm size", "Farm category" and "Region" as fixed factors. Due to the hierarchical structure, the variable "Veterinarian" is included in the model as a random factor. To describe "farm size", holdings are categorized into three groups by means of the 33%- and 67%-percentile of the number of livestock places per holding for 2011. The factor "Farm category" is based on the type of production type groups held per farm. Category "Breeding" comprises holdings with sows and piglets only, "Fattening" comprises holdings with weaners and/or fattening pigs only, and the category "Combined" holdings comprises weaners and/or fattening pigs combined with sows and piglets. The category "Changer" represents the holdings that stops keeping one group, kept an additional group or changes the production type group kept over time during the observational period (2011, 2013 and 2014). For the factor, "Region", the examined animal husbandry collective is divided into geographical areas based on agricultural structures in Germany [34].

Antibiotic use is measured twice yearly, resulting in up to six repeated measurements per holding. Mixed models are used for the analyses to account for the hierarchical data structure [35, 36]. Because of the non-equidistant time points, a flexible correlational structure between measurements of one holding is chosen. In addition, a compound symmetry structure is used to model the random veterinarian effect. Since the TF is not normally distributed, various regression models were adapted, and a model is selected based on the distribution of residuals. Due to its non-negative right-skewed distribution, we compare the following models: a negative binomial regression model and mixed models with three different transformations of TF (square root transformation, logarithm transformation after adding 0.1, and logarithm transformation after adding 1). For each production type group, different decisions are made within the model selection process. The distributions of the residuals of multi-factorial models with different transformations for the treatment frequency are provided as supplementary data (S1–S12 Figs).

The analyses were performed using the procedures GLIMMIX and MIXED in SAS, version 9.3, TS level 1M2 (SAS Institute Inc., Cary, NC, United States). To describe the influencing factors’ effects on the TF, the back-transformed estimators of the least-squares means with their associated 95% confidence intervals are considered for each category of factors. F-tests are used to assess the statistical significance of the fixed effects, considering p-values below 5% as statistically significant. The impact of the veterinarian random effect is analyzed using a likelihood ratio chi-square test comparing the full model with the reduced model, thus omitting the hierarchical level.

## Results

### Description

#### Study population

In total, 51 311 ADFs from participating pig farms were analyzed. For weaners and fattening pigs, the median was 4 antibiotic substance prescriptions per holding biannually (IQR = 1–13 prescriptions per holding for weaners and IQR = 1–10 prescriptions per holding for fattening pigs), while for sows and piglets the median were 6 and 7 prescriptions per holding (IQR = 2–14 prescriptions per holding for sows and IQR = 2–16 prescriptions per holding for piglets), respectively.

The number of participating holdings within the analyses increased over time. In the first half of 2011, 755 holdings were integrated into the evaluation, and 1 102 holdings were analyzed in the second half of 2014. The population composition changed only slightly relative to "Farm size", "Farm category" and "Region". Sow, piglet and weaner holdings comprised approximately 20% of these holdings each, while fattening pig holdings comprised approximately 40%. The increased number of evaluable fattening pig holdings per half-year was slightly more than that of other groups (see Table 1).

**Table 1:** Biannual treatment frequency distribution for sows, piglets, weaners and fattening pigs.

Half-year	Number of holdings	Semi-annual Treatment Frequency						
		Minimum	5%-quantile	25%-quantile	Median	75%-quantile	95%-quantile	Maximum
		**Sows**						
**2011–1**	149	-	-	0.2	1.2	5.0	29.9	46.4
**2011–2**	147	-	-	0.3	1.0	3.9	12.6	35.8
**2013–1**	142	-	-	0.3	1.0	5.4	22.4	53.6
**2013–2**	136	-	-	0.2	1.1	4.5	26.9	50.4
**2014–1**	167	-	-	0.3	1.2	3.5	21.4	46.5
**2014–2**	188	-	-	0.3	1.2	4.9	22.0	58.6
		**Piglets**						
**2011–1**	142	-	-	1.1	3.8	10.6	18.8	38.6
**2011–2**	141	-	-	0.8	3.9	12.4	26.5	45.0
**2013–1**	141	-	-	1.6	4.7	9.9	20.7	56.0
**2013–2**	134	-	-	0.6	2.7	5.8	14.5	40.1
**2014–1**	168	-	-	0.4	2.1	5.3	13.0	43.2
**2014–2**	191	-	-	0.2	1.7	4.5	11.1	48.6
		**Weaners**						
**2011–1**	141	-	-	1.6	10.0	31.3	98.4	138.8
**2011–2**	141	-	-	0.9	8.2	28.3	71.9	148.7
**2013–1**	149	-	-	0.2	9.3	26.8	90.3	159.7
**2013–2**	146	-	-	-	9.2	34.4	96.1	147.4
**2014–1**	188	-	-	1.7	12.2	37.9	89.2	135.3
**2014–2**	201	-	-	-	8.3	28.0	83.3	142.9
		**Fattening Pigs**						
**2011–1**	323	-	-	0.2	5.1	15.4	41.4	76.0
**2011–2**	320	-	-	0.4	5.3	15.0	42.4	76.0
**2013–1**	395	-	-	0.1	2.6	11.8	27.2	71.6
**2013–2**	411	-	-	0.1	2.7	9.9	29.1	72.8
**2014–1**	502	-	-	0.0	1.2	7.9	23.2	61.5
**2014–2**	522	-	-	0.1	0.7	6.7	20.9	56.8

-: observed zero; 0.0: zero by rounding

#### Antibiotic usage and treatment frequency

To describe the data basis of further statistical analyses, Table 1 shows the distribution of the biannual TF for the four production type groups. Consequently, following statements refer to a mere description of the data. In general, the median semi-annual TF constantly decreased from the first half of 2011 until the second half of 2014. This reduction is most noticeable in the fattening pigs, where the median TF decreased from 5.1 (in the first half of 2011) to 0.7 (in the second half of 2014) and furthermore in the piglets, where the median semi-annual TF decreased from 3.8 in the first half of 2011 to 1.7 in the second half of 2014. This is a TF reduction of more than 55% in piglets and 86% in fattening pigs within four years. The descriptive results of sow and weaner TF deviated. The median of both groups varied only slightly over time. For sows, the median TF remained between 1.0 and 1.2. For weaners, the values vary at higher levels between 8.2 and 12.2.

**Fig 1:**
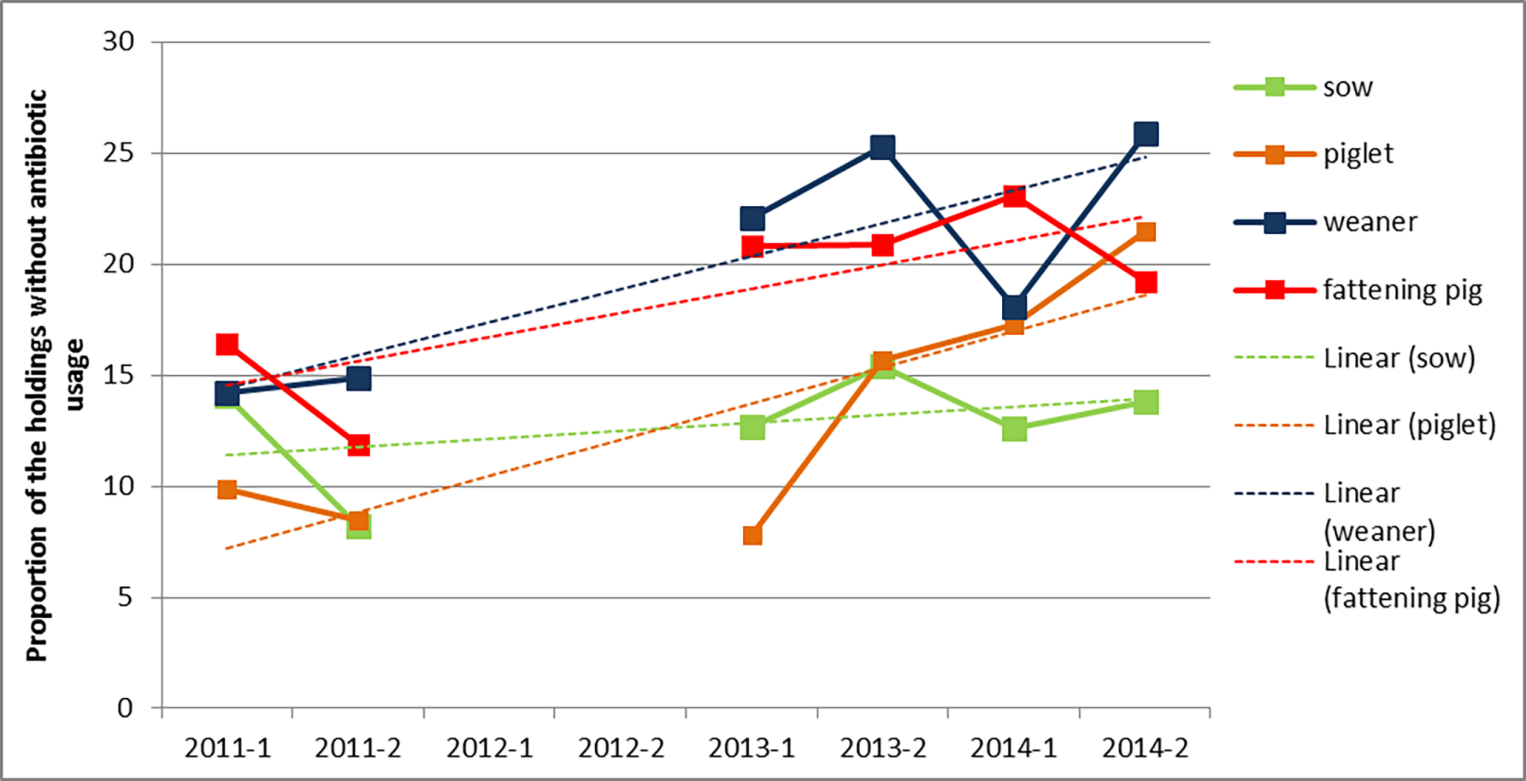
Percentage and trend line for participating farms with no antibiotic use by half-year for each production type group.

Fig 1 illustrates descriptive results about the change over time in the proportions of participants without antibiotic usage among the groups. The most obvious change occurred in the piglets. From less than 10% of holdings without antibiotic usage, their proportion increased constantly up to 21.5% in the second half of 2014. In the remaining groups, proportions varied: in 2013 and 2014, weaners and fattening pigs showed higher percentages of holdings without antibiotic use than in 2011. However, in 2013 and 2014 values varied between 18% and 26% for weaners and between 19% and 23% for fattening pigs. The proportion of sow holdings without antibiotic use always remained under 15%.

### Regression models

To improve the statistical significance of the data Tables 2–5 show the regression model results for each production type group. The estimates of fixed effects regression coefficients and random effects covariance parameters are provided in the supplementary material (S1 and S2 Tables). Within the model selection process, for groups with lower TFs, the highest goodness of fit was seen using the logarithm transformation after adding 0.1 (S1–S12 Figs). This occurred in the sow data with medians between 1.0 and 1.2 (Table 1). For distributions with a slightly higher average antibiotic use, the best model fit is obtained using the square root transformation. This applied to the distribution of the residuals of the TF in piglets (median from 1.7 to 4.7), weaners (median from 8.2 to 12.2) and fattening pigs (median from 0.7 to 5.3).

**Table 2:** Multi-factorial model results with logarithm transformation for the treatment frequency in sows.

Factor	Category	N	Mean	CI_l	CI_u	F-value	p-value
**Half-year**	global					1.597	0.165
	2011–1	149	1.087	0.699	1.664		
	2011–2	147	0.877	0.573	1.317		
	2013–1	142	0.827	0.530	1.263		
	2013–2	136	0.741	0.471	1.139		
	2014–1	167	0.723	0.477	1.074		
	2014–2	188	0.848	0.557	1.270		
**Farm size**	global					6.838	0.003
	lower third	221	0.529	0.302	0.884		
	middle third	365	0.859	0.541	1.335		
	upper third	343	1.292	0.856	1.926		
**Region**	global					0.010	0.990
	Middle	403	0.845	0.569	1.235		
	Northwest	469	0.863	0.561	1.303		
	East	57	0.823	0.320	1.925		
**Farm category**	global					0.450	0.642
	breeding	169	0.789	0.486	1.251		
	combined	563	0.945	0.632	1.390		
	changer	197	0.804	0.427	1.450		

CI_l, CI_u: Lower and upper limit of the 95% confidence interval

**Table 3:** Multi-factorial model results with square root transformation for the treatment frequency in piglets.

Factor	Category	N	Mean	CI_l	CI_u	F-value	p-value
**Half-year**	global					10.598	< .001
	2011–1	142	4.220	3.041	5.593		
	2011–2	141	4.634	3.319	6.167		
	2013–1	141	4.187	3.083	5.460		
	2013–2	134	2.303	1.565	3.183		
	2014–1	168	2.076	1.370	2.928		
	2014–2	191	1.648	1.038	2.397		
**Farm size**	global					20.676	< .001
	lower third	278	1.577	0.883	2.471		
	middle third	311	3.175	2.178	4.360		
	upper third	328	4.884	3.742	6.177		
**Region**	global					1.980	0.178
	Middle	388	3.536	2.639	4.562		
	Northwest	475	2.630	1.822	3.585		
	East	54	3.047	1.387	5.353		
**Farm category**	global					4.925	0.016
	breeding	157	2.599	1.667	3.737		
	combined	560	2.487	1.727	3.386		
	changer	200	4.237	2.791	5.983		

CI_l, CI_u: Lower and upper limit of the 95% confidence interval

**Table 4:** Multi-factorial model results with square root transformation for the treatment frequency in weaners.

Factor	Category	N	Mean	CI_l	CI_u	F-value	p-value
**Half-year**	global					5.580	< .001
	2011–1	141	18.417	12.527	25.437		
	2011–2	141	14.735	9.663	20.873		
	2013–1	149	18.209	12.684	24.731		
	2013–2	146	21.423	15.030	28.945		
	2014–1	188	23.109	16.909	30.275		
	2014–2	201	17.201	12.055	23.259		
**Farm size**	global					9.142	< .001
	lower third	279	13.948	8.729	20.385		
	middle third	268	17.481	11.521	24.678		
	upper third	419	25.749	19.012	33.507		
**Region**	global					8.487	0.005
	Middle	391	11.366	7.535	15.982		
	Northwest	529	11.359	7.394	16.173		
	East	46	39.043	21.962	61.005		
**Farm category**	global					9.400	< .001
	fattening	259	25.786	18.567	34.188		
	combined	532	13.715	9.239	19.072		
	changer	175	17.714	10.413	26.944		

CI_l, CI_u: Lower and upper limit of the 95% confidence interval

**Table 5:** Multi-factorial model results with square root transformation for the treatment frequency in fattening pigs.

Factor	Category	N	Mean	CI_l	CI_u	F-value	p-value
**Half-year**	global					17.541	< .001
	2011–1	323	5.090	3.636	6.787		
	2011–2	320	5.454	3.960	7.186		
	2013–1	395	3.911	2.731	5.301		
	2013–2	411	3.372	2.297	4.654		
	2014–1	502	2.294	1.452	3.329		
	2014–2	522	2.016	1.235	2.987		
**Farm size**	global					3.540	0.034
	lower third	534	2.782	1.703	4.123		
	middle third	893	3.741	2.572	5.129		
	upper third	1046	4.276	3.032	5.733		
**Region**	global					3.013	0.074
	Middle	1035	2.517	1.732	3.448		
	Northwest	1375	2.713	1.815	3.791		
	East	63	5.935	2.950	9.953		
**Farm category**	global					4.273	0.022
	fattening	1921	4.623	3.467	5.944		
	combined	332	3.100	1.943	4.527		
	changer	220	3.095	1.618	5.046		

CI_l, CI_u: Lower and upper limit of the 95% confidence interval

During the observational period, significant changes occurred in the TF among all groups; however, for sows, the mean TF values estimated via the model remained constant between 0.7 and 1.1. For weaners, the mean values are estimated between 14.7 and 23.1. Although the p-value shows a global statistical significance, no general trend is observed. In contrast, the estimated means are significantly reduced in the piglets and fattening pigs.

"Farm size" has a significant impact on the TF in all models. The estimated TF means increased with increasing size. For all groups in the category, "Upper third", the estimated mean is at least twice as high as for the "Lower third".

The factor "Region" has no significant impact on any group’s TF, except in the weaner model, since the category "East" has a nearly four-fold higher estimated mean (39.0) compared with the "Northwest" and "Middle" regions (11.4).

The factor "Farm category" significantly affects the TF in piglets, weaners and fattening pigs, while the sow model is unaffected. In piglets, the model estimated similar means in the categories, "Breeding" and "Combined", at 2.6 and 2.5. The category "Changer" has a significantly higher estimated mean of 4.2. The estimated means of the weaners and fattening pigs in the category "Fattening" are significantly higher (25.8 and 4.6) than in the categories "Combined" (13.7 and 3.1) and "Changer" (17.7 and 33.1).

In investigating the effect of veterinarians, values below 0.05 were observed for all p-values in the likelihood ratio test for each group model. This indicates that models addressing the veterinarian clusters fit the data better than models omitting the random veterinarian effect.

A sensitivity analysis to investigate the results’ stability was performed by omitting the cases where one veterinarian ID is connected with only one farm ID (data not shown). The analysis showed no changes in interpreting of the p-values that assessed the fixed effects and the veterinarian random effect for all group models.

## Discussion

This longitudinal study used data from mandatory ADF documentation in Germany. Veterinarians and farmers participated voluntarily in a panel study and provided data from 2011, 2013, and 2014. Based on the calculated semi-annual TF for each pig holding, the influence of various factors on antibiotic consumption on pig farms was analyzed, using regression models. This study’s main achievement is observing a cohort of pig holdings over several years.

This study is a longitudinal extension of the cross-sectional VetCAb project. Van Rennings et al. [11] showed the successful implementation of a monitoring system for antibiotic use by presenting the first results representative for German pig holdings. To transfer this knowledge, the study design concerning data collection and analysis as well as the general measurement of antibiotic usage was adopted. Furthermore, recruitment focused on maintaining the pilot collective to minimize a possible selection and migration bias.

The data presented here are based on voluntary participation, thus carrying the risk of selection bias, or migration bias in longitudinal studies. The true antimicrobial use may have been higher and the reduction less pronounced than that in the presented data. However, the TF range results suggest that both selection bias [11] and migration bias are unlikely.

Additionally, a bias due to ADF misallocation must be discussed. Using ADFs as data sources risks assigning the ADFs and antibiotic treatments to the incorrect production type groups. The group designations were not standardized, and were often used in colloquial speech for the ADFs, resulting in the allocations being ambiguous. Specifically, the term "weaner" has various meanings in veterinary and farming practice in Germany. Therefore, an exalted misallocation frequency is possible; however, we assume that veterinarians will define production types homogeneously by adhering to the Medicinal Products Act, so that this effect will decrease over the years.

In this study, antibiotic use is measured by the treatment frequency calculated based on the nUDD. Most other studies on antibiotic consumption in veterinary medicine are based on sales data (viz. amounts). Merle et al. [9], van Rennings et al. [27] and Schaekel et al. [29] described the differences between UDD and DDDA in detail. Here, documenting data for calculating used daily doses (UDD) offers two practical advantages. First, in contrast to the theoretical DDDA (defined daily dose for animals), which estimates the use based on the sales data or amounts applied, nUDD gives direct insight into the antibiotic consumption on site. The key differences are that the TF (nUDD) provides information on the actual number of animals treated, and, if the total amount of antimicrobials used is recorded, allows assessing the actual dose of the active ingredient used. If DDDA is applied, standard dosages and standard animal weights are used instead, which will thus provide inaccurate information when animals are treated during different live periods. Consequently, the results are observed with additional variability and therefore may lead to diluted effects when comparing TFs. Thus, calculations based on UDD, especially for running risk factor models, may achieve less biased results. Because the number of treated animals and treatment days are documented on the ADFs in Germany, the nUDD can be calculated directly, without the quantity of active substances or standardized animal weights, so that these possible sources of error remain accurate. Second, the production type of the treated animal (group) is usually indicated on the ADFs in Germany; therefore, using the ADFs facilitates associating the antibiotic consumption with the group treated.

Although TF is similar to amount-based calculations for antibiotic use, its comparison to other studies is restricted due to systematically different approaches in defining standard weights and DDDA. In addition, this comparison is hindered by varying group definitions. In the present VetCAb study, the observed animal holdings are divided into the groups piglet, weaner, fattening pig and sow. In other surveys, production type groups were summarized, such as those of Bos et al. [37] and Jensen et al. [38], who analyzed piglets and sows together in one group. Conversely, some document no group separation at all, such as in Hosoi et al. [39]. Even if production types are separated into groups, the definitions of these groups may differ, such as in the surveys of Callens et al. [40], Trauffler et al. [28] and Sjölund et al. [41]. Hence, it is crucial to differentiate antibiotic use data and standardize definitions when comparing data between studies, regions, and countries.

Overall, internationally and even nationally, no harmonized approach exists for assessing antibiotic consumption; therefore, increased attention should be paid to standardizing definitions and calculation methods in antibiotic monitoring to compare antibiotic use.

Despite the many differences described above, temporal trends within the systems can be compared. In this study, sufficient data were available to assess the temporal development of antibiotic usage in all animal groups. While the median TF for sows remained unchanged and constantly low over the study period, piglets and fattening pigs showed remarkably reduced antimicrobial consumption. In contrast, antibiotic use in weaners fluctuated significantly, which requires further investigation.

In general, the marked differences in the TFs between groups may be explained by animals’ different exposures to infectious hazards. Since fattening animals (piglets, weaners and fattening pigs) receive the most antibiotics in pig farming, this area should be the focus for reduction. The fluctuating TF medians in the weaner collective were striking, which could be explained by the possible misallocation described above.

In total, a statistically significant reduction in antibiotic use over time is evident in this study. This is consistent with reported sales data in Germany, where since 2011, a reduction by 468 tons to approximately 1 238 tons was observed in 2014 [12]. This trend appears to continue in the following years. In the official monitoring system, the median and the upper quartile of the TF decreased constantly. For weaners (up to 30 kg bodyweight (BW)) and fattening pigs (from 30 kg BW), the medians decreased from 4.8 and 1.2, respectively, in the second half of 2014 to 3.4 and 0.4, respectively, in the first half of 2016 [14, 15]. In the QS system, the therapy index medians decreased from 10.71 (weaners, up to 30 kg BW) and 1.76 (fattening pigs, from 30 kg BW), respectively, in the second half of 2014 to 3.53 and 0.37, respectively, in the first half of 2016 [13].

The reduced antibiotic usage can also be seen in other European countries, as shown in Sjölund et al.’s study [42]. The sixth European Surveillance of Veterinary Antimicrobial Consumption (ESVAC) report [19] showed lower sales of veterinary antimicrobial agents for food-producing species per population corrected unit (in mg/PCU) in 2014 for all animals in countries including Belgium, the Netherlands, Sweden and Germany compared to former years. Austria, Denmark, Poland and the UK also reduced their veterinary antimicrobial agent sales for food-producing species per population corrected unit, but in these countries, the reductions began later (in 2012 and 2013, respectively) [19]. In Denmark, the total amounts of antibiotic substances as well as the DAPD (DDDA per 1 000 animals per day), increased from 2011 to 2013 in all groups, until the DAPD decreased in 2014 [43]. However, antibiotic use in Denmark was significantly reduced a few years prior; compared with 2009, the DAPD decreased in 2011 in all groups. This was related to the yellow card initiative, a benchmarking system for pig farms established by the Danish Veterinary and Food Administration (DVFA) in 2010 [44]. In the Netherlands, the reduction in antimicrobial use began in 2007 and has continued since [16].

In summary, nearly all countries with antibiotic use monitoring systems in livestock husbandry document success in reducing antibiotic consumption for many reasons. One of the main reasons is that awareness in both society and the agricultural sector has improved. This increase developed due to implementing monitoring systems, publishing scientific studies from across Europe (e.g., [28, 37, 45–50]) and various measures taken by international and national policy makers [19, 51–53].

International surveys show that antibiotic use in animal husbandry cannot be reduced indefinitely. In 2015, the National Veterinary Institute of Sweden (SVA) published the yearly Swedish Veterinary Antibiotic Resistance Monitoring (SVARM) results, which showed that the temporal trend of veterinary antimicrobial consumption in Sweden has reduced over several years. In contrast, antibiotic sales for pigs remained stagnate over the past five years. However, another change occurred instead. Product sales for individual medications increased, while sales of group medications decreased [17]; thus, it appears that more individual and less metaphylactic treatments have occurred.

Diseases requiring antibiotic treatment can occur in any animal holding type. For this reason, the aim cannot be to ban antibiotic uses, but to change its application. Responsible antibiotic handling does not mean non-use, but prudent use. It must be assumed that in the future, a plateau will be reached, and the reduction will stagnate at the necessary level. Further studies are needed to assess the antimicrobial level needed to treat diseased animals without conflicting with animal welfare through the legal compulsion for further reductions.

Furthermore, all measures must be evaluated regularly and in detail to adjust the monitoring and benchmarking system if necessary. As a first step, multiple linear regression for each defined production type group was performed to investigate weather monitoring antibiotic use should be accompanied by evaluating secondary data.

In this study, the estimated means of TF rose with increasing farm size in all production type groups. In contrast, Vieira et al. [54] identified higher TF on small farms and mentioned worse hygiene management as a possible reason. Van Rennings et al. [11] also obtained different results. The authors determined that farm size did not significantly impact the TF, except for a slight influence in the weaner model. Considering that part of van Rennings et al.’s study population [11] was also analyzed in this study, this influence appears to have changed over time, as the extended study population and more detailed model may have influenced the recent study. Van der Fels-Klerx et al. [25] noted an influence of farm size on antibiotic use in their study population in 2011. They suggested that a rising probability of infection with an increased number of animals could be responsible for this and refer to Österberg et al. [22], Hautekiet et al. [23] und García-Feliz et al. [24]. Regarding the link between herd size and antimicrobial resistance, various patterns are also described [55].

In our study, spatial factors did not significantly impact the TF, except for in weaners. This result is consistent with those of van Rennings et al. [11]. Although a high density of pig holdings prevails in some regions, and the presumption suggests higher infection pressure due to the proximity of neighboring stables [56], high farm density and high antibiotic use were not significantly associated in this study. Even in the weaner model, a statistically significant higher TF was determined in the "East" region, which comprises few but large farms [34].

The factor "Farm category" has a significant effect on the TF in piglets, weaners and fattening pigs, while the sow model was unaffected. Fattening pigs show similar results to those of van der Fels-Klerx et al. [25]; on specialized fattening farms, significantly more antibiotics were used than on combined farms / farrow-to-finish farms. This finding corresponds to the assumption that increased animal movement and pooling of animals from different stables, and thus of different farm-specific germ spectra, results in an increased risk of infection. These results concur with those published by Casal et al. [57] and Moreno [58]. The same applies to the weaners. In sow holdings, the farm category has no significant influence on the TF, whereas in piglet holdings, a significant impact could be seen, but neither "Combined" nor specialized "Breeding" farms used considerably more antibiotics. (In the piglet group, the significance of the factor seems to be due to the high-level antibiotic usage in the "Changers" group. In this group, production system conversion leading to unstable production conditions may have influenced the disease incidence, leading to increased antibiotic use.) These findings support the above hypothesis; sows and piglets were not moved to other stables, regardless of farm category. The increased infection pressure is only apparent after the first regrouping. Van der Fels-Klerx et al. [25] has received other results in this regard; specialized sow farms used fewer antibiotics for piglets than did farrow-to-finish farms. They hypothesized that this result may be due to the term "piglet" is being used longer on farrow-to-finish farms than on specialized farms.

According to van Rennings et al. [11], the influence of veterinarian is taken into account within the adjusted analyses and has again a significant effect on the TF. This could be explained by the veterinarian’s different specializations or typical antimicrobial treatment routines (e.g., using combined products).

Overall, the factors, "Farm size", "Veterinarian" and "Farm category", appears to impact the TF, while the "Region" factor shows no effect when the data are adjusted for confounding. However, it has to be assumed that these three identified factors are not solely influential; thus, to improve existing systems for antibiotic monitoring or establish new intervention measures, additional studies are needed. Evaluating the secondary data may greatly benefit this.

## Conclusions

In the longitudinal VetCAb sentinel study, antibiotic administration decreased significantly over time in the study population. The factors, "Farm size", "Veterinarian" and "Farm category", appeared to impact the treatment frequency. Considering these effects, these factors should complement antibiotic use monitoring.

## Supporting information

S1 Fig Distribution of the residuals of the multi-factorial model with logarithm transformation after adding 0.1 for the treatment frequency in piglets. (TIF)

S2 Fig Distribution of the residuals of the multi-factorial model with logarithm transformation after adding 1 for the treatment frequency in piglets. (TIF)

S3 Fig Distribution of the residuals of the multi-factorial model with square root transformation for the treatment frequency in piglets. (TIF)

S4 Fig Distribution of the residuals of the multi-factorial model with logarithm transformation after adding 0.1 for the treatment frequency in sows. (TIF)

S5 Fig Distribution of the residuals of the multi-factorial model with logarithm transformation after adding 1 for the treatment frequency in sows. (TIF)

S6 Fig Distribution of the residuals of the multi-factorial model with square root transformation for the treatment frequency in sows. (TIF)

S7 Fig Distribution of the residuals of the multi-factorial model with logarithm transformation after adding 0.1 for the treatment frequency in weaners. (TIF)

S8 Fig Distribution of the residuals of the multi-factorial model with logarithm transformation after adding 1 for the treatment frequency in weaners. (TIF)

S9 Fig Distribution of the residuals of the multi-factorial model with square root transformation for the treatment frequency in weaners. (TIF)

S10 Fig Distribution of the residuals of the multi-factorial model with logarithm transformation after adding 0.1 for the treatment frequency in fattening pigs. (TIF)

S11 Fig Distribution of the residuals of the multi-factorial model with logarithm transformation after adding 1 for the treatment frequency in fattening pigs. (TIF)

S12 Fig Distribution of the residuals of the multi-factorial model with square root transformation for the treatment frequency in fattening pigs. (TIF)

S1 Table Estimates of regression coefficients for fixed effects in the multi-factorial models per production type. (DOCX)

S2 Table Estimates of covariance parameters for random effects in the multi-factorial models per production type. The coloured lines show the estimated variances for observations within one veterinarian or within one of the six time-points.
(DOCX)

## Abbreviations

ADF: Application and Delivery Form
BW: Body Weight
DAPD: DDDA per 1,000 Animals per Day
DDDA: Defined Daily Dose for Animals
ID: Identification Number
TF: Treatment Frequency
QS: QS Qualität und Sicherheit GmbH
(n)UDD: (Number of) Used Daily Dose(s)
VetCAb: Scientific Study "Veterinary Consumption of Antibiotics"
