# Supplementary figures and images for: Antibiotic use on German pig farms - A longitudinal analysis for 2011, 2013 and 2014
Source: PLoS One. 2018 Jul 3;13(7):e0199592. doi: 10.1371/journal.pone.0199592 (PMC6029768; doi:10.1371/journal.pone.0199592)

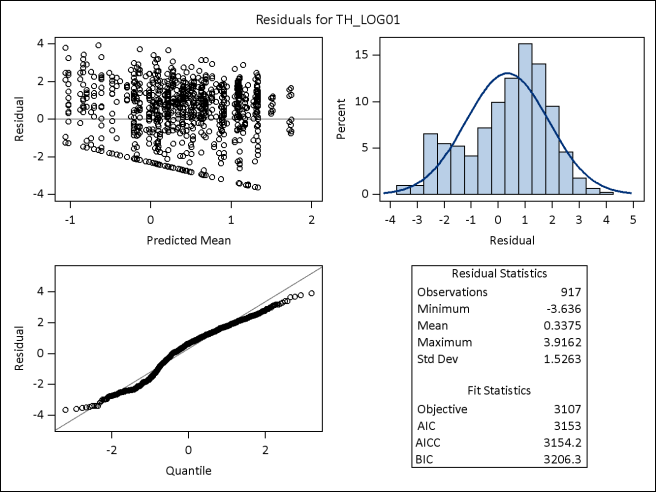

Supplement: S1 Fig — (TIF) [file pone.0199592.s001.tif]

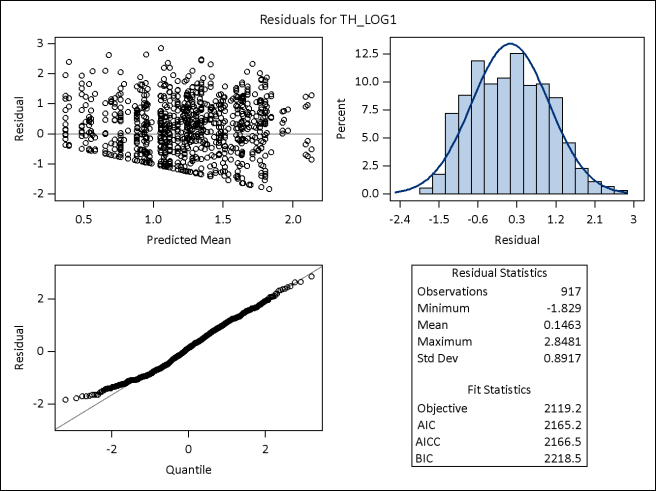

Supplement: S2 Fig — (TIF) [file pone.0199592.s002.tif]

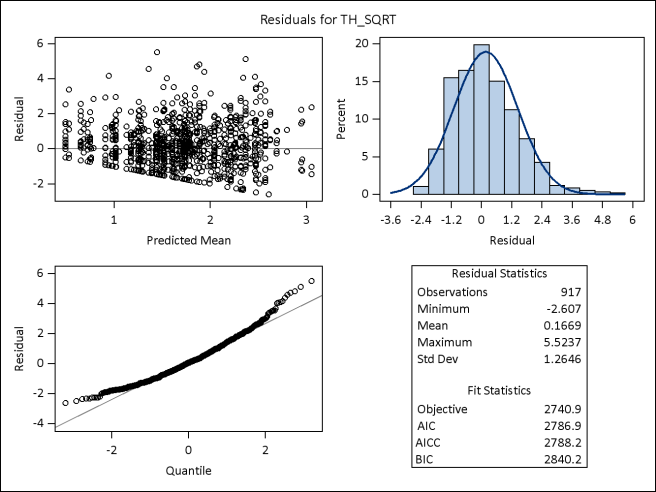

Supplement: S3 Fig — (TIF) [file pone.0199592.s003.tif]

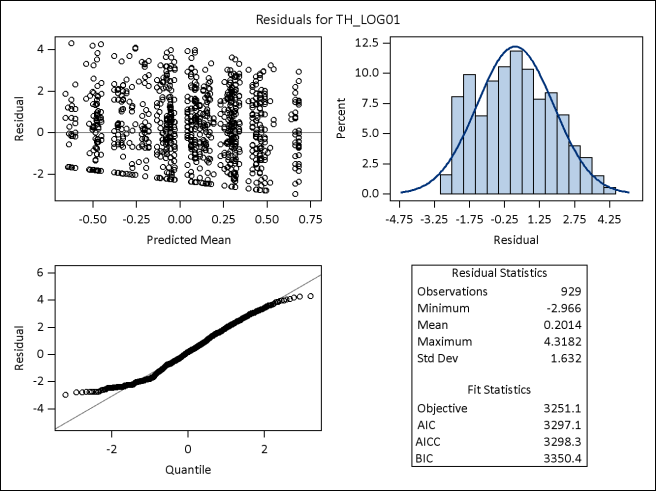

Supplement: S4 Fig — (TIF) [file pone.0199592.s004.tif]

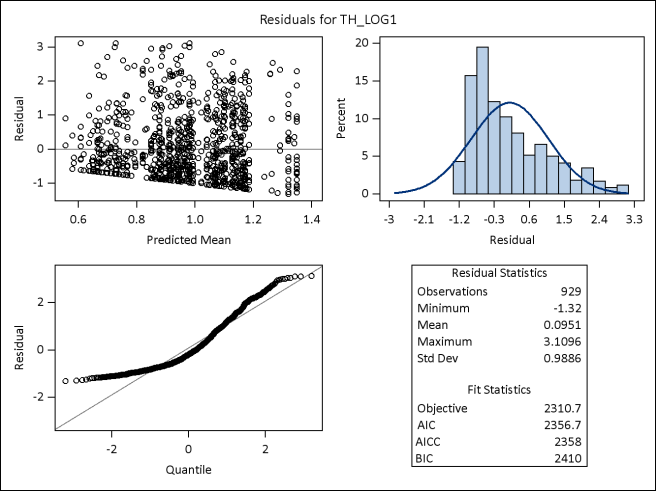

Supplement: S5 Fig — (TIF) [file pone.0199592.s005.tif]

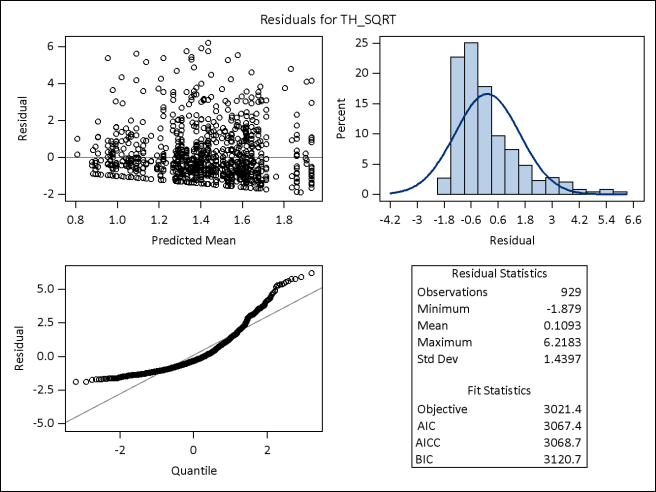

Supplement: S6 Fig — (TIF) [file pone.0199592.s006.tif]

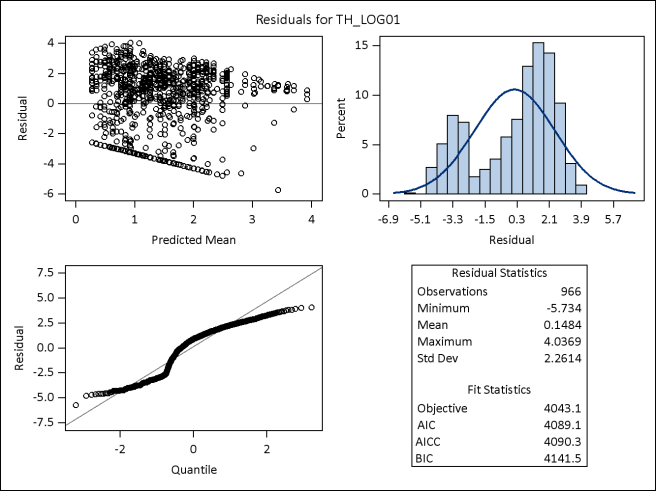

Supplement: S7 Fig — (TIF) [file pone.0199592.s007.tif]

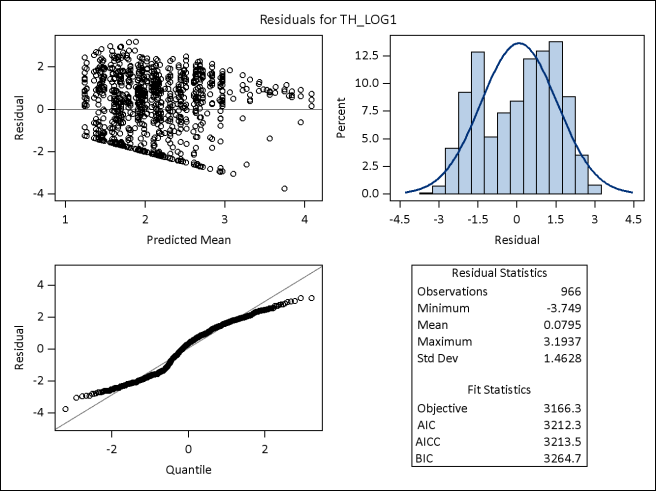

Supplement: S8 Fig — (TIF) [file pone.0199592.s008.tif]

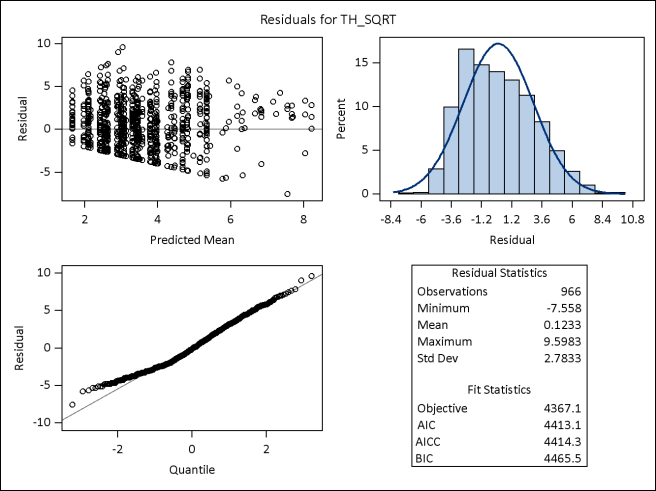

Supplement: S9 Fig — (TIF) [file pone.0199592.s009.tif]

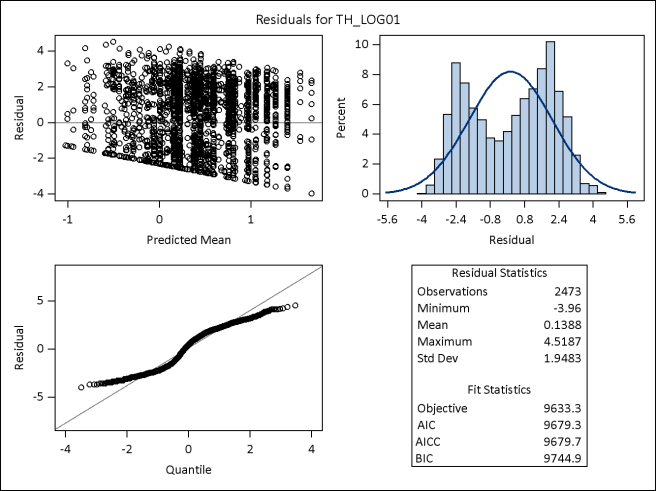

Supplement: S10 Fig — (TIF) [file pone.0199592.s010.tif]

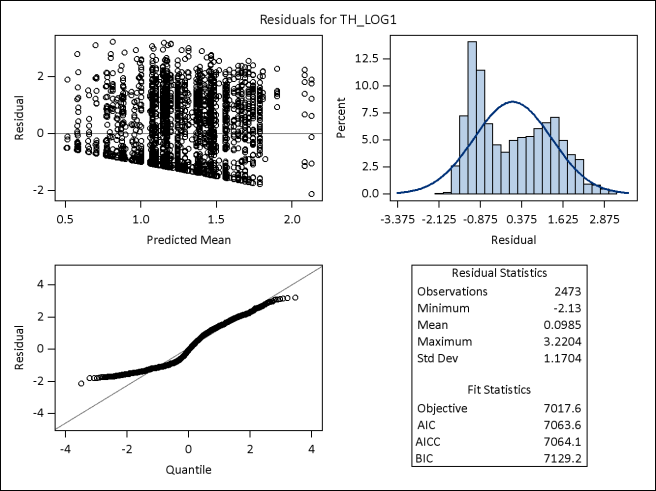

Supplement: S11 Fig — (TIF) [file pone.0199592.s011.tif]

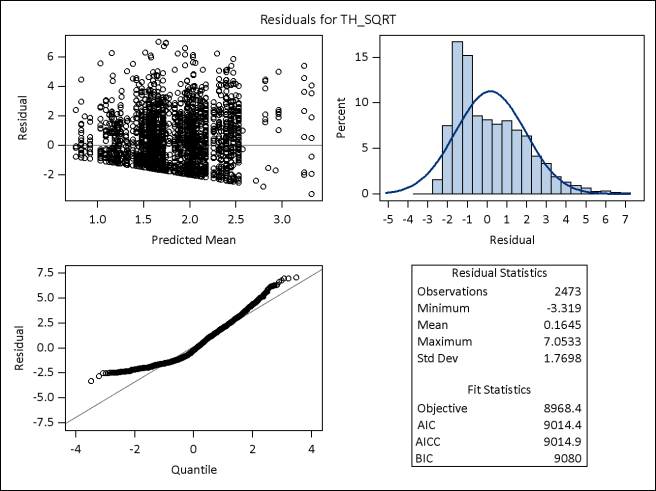

Supplement: S12 Fig — (TIF) [file pone.0199592.s012.tif]
