# Supplementary material for: Antibiotic use on German pig farms - A longitudinal analysis for 2011, 2013 and 2014
Source: PLoS One. 2018 Jul 3;13(7):e0199592. doi: 10.1371/journal.pone.0199592 (PMC6029768; doi:10.1371/journal.pone.0199592)
Supplement: S1 Table — (DOCX) [file pone.0199592.s013.docx]

S1 Table. Estimates of regression coefficients for fixed effects in the multi-factorial models per production type

|  |  | **Sows^1^** | | **Piglets^2^** | | **Weaner^2^** | | **Fattening pigs^2^** | |
| --- | --- | --- | --- | --- | --- | --- | --- | --- | --- |
| **Factor** | **Category** | **Estimate** | **Std.error** | **Estimate** | **Std.error** | **Estimate** | **Std.error** | **Estimate** | **Std.error** |
| **Intercept** |  | 0.271 | 0.428 | 2.050 | 0.312 | 6.689 | 0.817 | 2.013 | 0.390 |
| **Half-year** | 2011-1 | 0.224 | 0.181 | 0.771 | 0.147 | 0.144 | 0.272 | 0.836 | 0.125 |
|  | 2011-2 | 0.029 | 0.159 | 0.869 | 0.162 | -0.309 | 0.260 | 0.915 | 0.121 |
|  | 2013-1 | -0.023 | 0.146 | 0.763 | 0.116 | 0.120 | 0.224 | 0.558 | 0.090 |
|  | 2013-2 | -0.120 | 0.139 | 0.234 | 0.084 | 0.481 | 0.235 | 0.417 | 0.081 |
|  | 2014-1 | -0.142 | 0.110 | 0.157 | 0.085 | 0.660 | 0.171 | 0.095 | 0.065 |
|  | 2014-2 (reference) | 0.000 |  | 0.000 |  | 0.000 |  | 0.000 |  |
| **Farm size** | lower third | -0.794 | 0.215 | -0.954 | 0.148 | -1.340 | 0.321 | -0.400 | 0.151 |
|  | middle third | -0.373 | 0.186 | -0.428 | 0.138 | -0.893 | 0.325 | -0.134 | 0.119 |
|  | upper third (reference) | 0.000 |  | 0.000 |  | 0.000 |  | 0.000 |  |
| **Region** | Middle | 0.024 | 0.371 | 0.135 | 0.269 | -2.877 | 0.715 | -0.850 | 0.346 |
|  | Northwest | 0.043 | 0.375 | -0.124 | 0.271 | -2.878 | 0.714 | -0.789 | 0.347 |
|  | East (reference) | 0.000 |  | 0.000 |  | 0.000 |  | 0.000 |  |
| **Farm category** | breeding / fattening | -0.016 | 0.289 | -0.446 | 0.212 | 0.869 | 0.460 | 0.391 | 0.211 |
|  | combined | 0.145 | 0.223 | -0.481 | 0.153 | -0.505 | 0.393 | 0.002 | 0.240 |
|  | changer (reference) | 0.000 |  | 0.000 |  | 0.000 |  | 0.000 |  |

^1^model with logarithm transformation for the treatment frequency

^2^model with square root transformation for the treatment frequency
