# Supplementary material for: Antibiotic use on German pig farms - A longitudinal analysis for 2011, 2013 and 2014
Source: PLoS One. 2018 Jul 3;13(7):e0199592. doi: 10.1371/journal.pone.0199592 (PMC6029768; doi:10.1371/journal.pone.0199592)
Supplement: S2 Table — The coloured lines show the estimated variances for observations within one veterinarian or within one of the six time-points. (DOCX) [file pone.0199592.s014.docx]

S2 Table. Estimates of covariance parameters for random effects in the multi-factorial models per production type. The coloured lines show the estimated variances for observations within one veterinarian or within one of the six time-points.

|  |  | **Sows^1^** | **Piglets^2^** | **Weaner^2^** | **Fattening pigs^2^** |
| --- | --- | --- | --- | --- | --- |
| **Covariance parameter** | **Subject** | **Estimate** | **Estimate** | **Estimate** | **Estimate** |
| **Variance** | veterinarian | 0.192 | 0.102 | 0.549 | 0.324 |
| **CS** | veterinarian | 0.055 | 0.029 | 0.433 | -0.042 |
| **UN(1,1)** | holding (veterinarian) | 2.833 | 1.777 | 7.482 | 3.592 |
| **UN(2,1)** | holding(veterinarian) | 1.073 | 1.034 | 3.164 | 1.889 |
| **UN(2,2)** | holding(veterinarian) | 2.011 | 2.433 | 5.319 | 3.622 |
| **UN(3,1)** | holding(veterinarian) | 0.773 | 0.694 | 2.691 | 1.154 |
| **UN(3,2)** | holding(veterinarian) | 0.431 | 1.180 | 1.333 | 1.643 |
| **UN(3,3)** | holding(veterinarian) | 2.658 | 1.869 | 7.001 | 3.178 |
| **UN(4,1)** | holding(veterinarian) | 0.902 | 0.052 | 1.074 | 1.046 |
| **UN(4,2)** | holding(veterinarian) | 0.709 | 0.212 | 2.507 | 1.319 |
| **UN(4,3)** | holding(veterinarian) | 1.706 | 0.818 | 5.663 | 1.687 |
| **UN(4,4)** | holding(veterinarian) | 2.777 | 1.085 | 10.379 | 2.897 |
| **UN(5,1)** | holding(veterinarian) | 1.144 | 0.066 | 1.758 | 1.019 |
| **UN(5,2)** | holding(veterinarian) | 1.004 | -0.134 | 1.649 | 1.042 |
| **UN(5,3)** | holding(veterinarian) | 1.378 | 0.745 | 4.158 | 1.440 |
| **UN(5,4)** | holding(veterinarian) | 1.482 | 0.762 | 5.452 | 1.442 |
| **UN(5,5)** | holding(veterinarian) | 2.278 | 1.282 | 7.779 | 2.317 |
| **UN(6,1)** | holding(veterinarian) | 0.113 | -0.065 | 3.561 | 0.581 |
| **UN(6,2)** | holding(veterinarian) | 0.720 | -0.365 | 2.781 | 0.875 |
| **UN(6,3)** | holding(veterinarian) | 0.983 | 0.448 | 3.092 | 1.021 |
| **UN(6,4)** | holding(veterinarian) | 1.267 | 0.655 | 4.479 | 1.199 |
| **UN(6,5)** | holding(veterinarian) | 1.429 | 0.673 | 4.869 | 1.258 |
| **UN(6,6)** | holding(veterinarian) | 2.610 | 1.253 | 7.327 | 2.259 |

^1^model with logarithm transformation for the treatment frequency

^2^model with square root transformation for the treatment frequency

CS: compound symmetry covariance structure

UN: unstructured covariance for repeated measurements in one holding, displayed are the estimated elements of the lower triangular matrix
